# Alkylphenols in Surface Sediments of the Gulf of Gdansk (Baltic Sea)

**DOI:** 10.1007/s11270-014-2040-8

**Published:** 2014-07-24

**Authors:** Iga Koniecko, Marta Staniszewska, Lucyna Falkowska, Dorota Burska, Joanna Kielczewska, Anita Jasinska

**Affiliations:** Institute of Oceanography, University of Gdansk, Al. Marszalka Pilsudskiego 46, 81-378 Gdynia, Poland

**Keywords:** 4-nonylphenol, 4-*tert*-octylphenol, Organic carbon, Black carbon in sediments, Gulf of Gdansk, Southern Baltic

## Abstract

The widespread use of alkylphenols in European industry has led to their presence in the environment and the living organisms of the Baltic Sea. The present study (2011–2012) was designed to determine the concentrations of alkylphenols, 4-nonylphenol (NP) and 4-*tert*-octylphenol (OP), in surface sediments of the Gulf of Gdansk, a section of the Baltic that lies in close proximity to industrial and agricultural areas and borders with an agglomeration of nearly one million inhabitants. It is also where the Vistula, the largest Polish river, ends its course. In spring, large concentrations of 4-nonylphenol and 4-*tert*-octylphenol were washed off into the coastal zone with meltwater. In summertime, sediments near the beach had the highest alkylphenol concentrations (NP—2.31 ng g^−1^ dw, OP—13.09 ng g^−1^ dw), which was related to tourism and recreational activity. In silt sediments located off the coast, the highest NP (1.46 ng g^−1^ dw) and OP (6.56 ng g^−1^ dw) amounts were observed in autumn. The origin of OP and NP at those test stations was linked to atmospheric transport of black carbon along with adsorbed alkylphenols.

## Introduction

4-nonylphenol (NP) and 4-*tert*-octylphenol (OP) belong to a category of compounds that are capable of mimicking the hormones of living organisms and thus disrupting their hormonal balance. They influence the synthesis, transportation, bonding, action or excretion of hormones that occur naturally in the system and are responsible for maintaining homeostasis as well as for reproduction or the behaviour of a living organism (US EPA 2010).

Phenol derivatives can imitate the action of the sex hormone 17β-estradiol. The estrogenicity of alkylphenols was discovered as early as the 1930s. First tests to be conducted by Dodds and Lawson in 1938 showed that a 100-mg dose of 4-propylphenol administered to female rats with surgically removed ovaries resulted in changes in their reproductive system that were characteristic of the estrous cycle, despite the fact that they were lacking in estradiol (Markey et al. 2001). NP exhibits an endocrine-disrupting action which is three times as strong as DDT. Additionally, 4-nonylphenol and 4-*tert*-octylphenol are both acutely toxic to fish, invertebrates and algae (Servos 1999; Flint et al. 2012).

Due to their lipophilic characteristics, 4-nonylphenol and 4-*tert*-octylphenol can combine with both live and dead organic matter in water and in bottom sediments. According to a research carried out by Ying et al. (2003), 4-*tert*-octylphenol and 4-nonylphenol have the highest degree of sorption on sediment particles compared to bisphenol A and hormones such as 17β-estradiol (E2) and 17α-ethinyl estradiol (EE2).

Alkylphenyl ethoxylates (precursors of alkylphenols) have been used in industry for over 50 years. Nearly 80 % of all alkylphenyl etoxylates currently produced are nonylphenol etoxylates. 4-nonylphenol is used mainly in the production of surface active agents, while 4-*tert*-octylphenol is used for the production of synthetic materials (HELCOM 2011). Alkylphenols 100,000 tonnes are produced in Europe every year, accounting for one-third of the global production, and in 2001, EU production of 4-*tert*-octylphenol amounted to 23,000 tonnes. There are 12 major producers of octylphenols in Europe. Russia produces 17,000 tonnes of octylphenol every year (HELCOM 2011). The main producer of nonylphenol in Poland is PCC Synteza SA in Kedzierzyn-Kozle (about 12,000 tons every year).

The Gulf of Gdańsk, situated in the south part of the Baltic Sea, is exposed to contaminants like alcylphenols (Staniszewska and Falkowska 2011; Staniszewska et al. 2014). There is very little available information on alkylphenol concentrations in the sediments of the Baltic Sea. According to HELCOM, the highest OP and NP concentrations were detected in the central area of the Baltic and in the Danish straits region, where concentrations exceeded 10.2 ng g^−1^ dw (OP) and 540 ng g^−1^ dw (NP), whereas the lowest concentrations of both alkylphenols were found in the sediments of the Gulf of Finland and Gulf of Bothnia (HELCOM 2010).

The aim of the present study was to establish the concentrations of 4-nonylphenol and 4-*tert*-octylphenol in the surface sediments of the Gulf of Gdansk as well as to determine the spatial and temporal variability of the compounds’ concentrations. A particular focus was placed on indicating potential external sources of alkylphenols in the Gulf of Gdansk, hence the situation of test stations at sea along the coastline from the estuary of the Vistula river, the second largest river in the Baltic drainage basin, and close to the outlets of several smaller rivers measuring between 10 and 20 km each. The possibility of alkylphenols arriving to the Gulf of Gdansk via atmospheric transportation from local and distant sources was analysed, taking into account the presence of organic carbon (OC) and black carbon (BC) in surface sediments.

## Materials and Methods

### Sample Collection

**Fig. 1:**
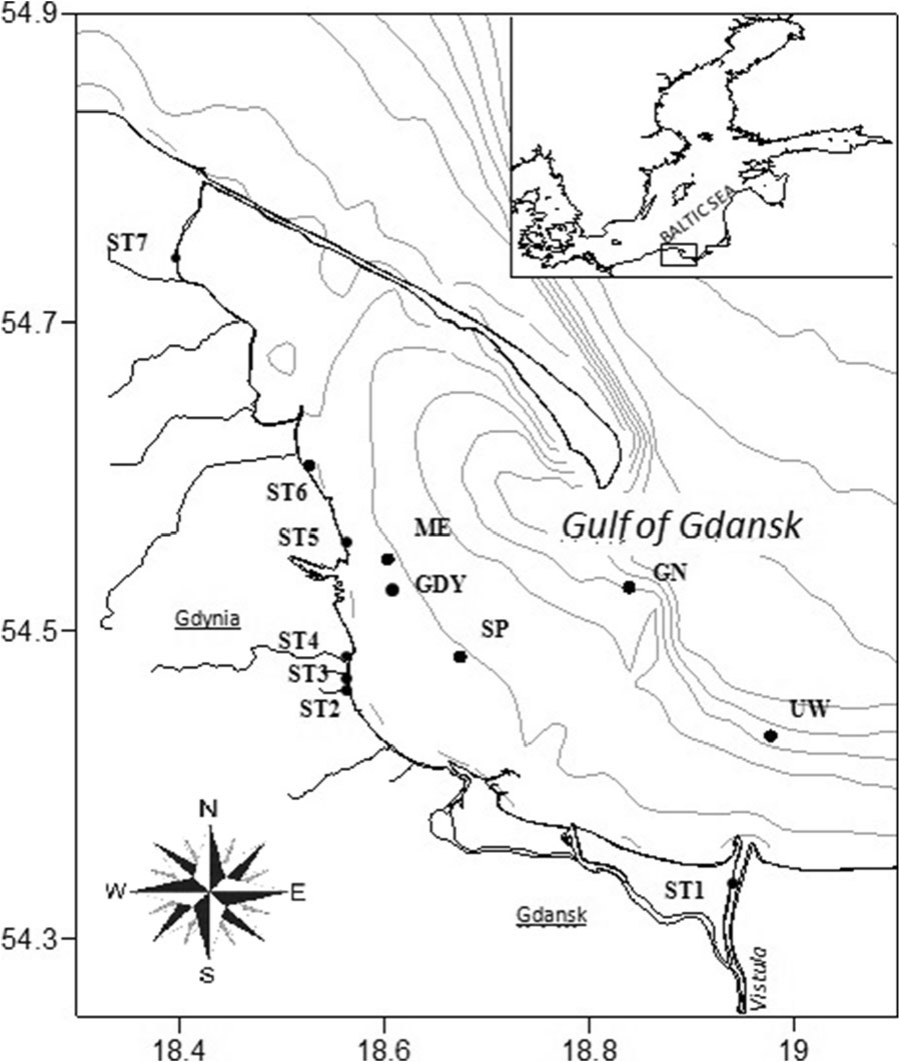
The locations of sediment sampling sites in the Gulf of Gdansk in 2011–2012

Sediment samples were collected in 2011 and 2012 in three different seasons (spring, summer and autumn). Seven coastal stations were used, and, of these, three were located by river outlets: the Vistula (ST1), the Kacza (ST2) and the Gizdepka (ST6), while the other four were situated near urbanised areas: Orlowo Pier (ST3), the Seaside Boulevard in Gdynia (ST4), Mechelinki (ST5), Swarzewo (ST7). Five more stations were located at sea further away from the coastline, below the 4-m isobath: ME, GDY, SP, GN, UW (Table 1) (Fig. 1).

**Table 1:** The characteristics and location of sediment sample collection sites

Station symbol/Station name		Characteristics	Location
ST1	Vistula Swibno	The Vistula is the main source of pollutants flowing from the whole of Poland into the Baltic Sea. Measuring 1,047 km, it originates in the mountains at a height of 1,107 m above sea level. The drainage area of the Vistula is 194,424 km^2^. The average volume of waters introduced into the Gulf of Gdansk is 1,046 m^3^ s^−1^.	Pontoon bridge on the Vistula
ST2	Kacza River	The river flows through Gdynia (14.8 km). Drainage area (53.8 km^2^) densely covered with bushes and woods interspersed with small areas of marshy meadows and peatland. The main sources of pollutants are the nearby urbanised areas.	50 m ahead of the river outlet
ST3	Orlowo Pier	Wooden pier located in Gdynia by the Kacza river outlet and close to recreational beaches.	Sea coast
ST4	Gdynia Seaside Boulevard	The Seaside Boulevard, adjacent to a marina and the city beach of Gdynia, is a recreational site, frequented by tourists and locals alike.	Sea coast
ST5	Mechelinki	Recreational site, the beach in Mechelinki.	Sea coast
ST6	Gizdepka River	The river flows in a narrow ravine across agricultural areas. The main source of pollution is surface run-off from cultivated fields and farms. The river, measuring 11.8 km, ends its course in Puck Bay near Oslonino.	River outlet
ST7	Swarzewo	Holiday destination on Puck Bay, featuring a water purification plant.	Sea coast
UW		Station located 7 km to the North-East of the Vistula estuary, 40 m below sea level.	54° 25.90′ N 18° 58.80′ E
SP		Station located 7 km away from Orlowo Pier, 17 m below sea level.	54° 28.91′ N 18° 40.43′ E
GDY		Station located 4 km away from the entrance to Gdynia Harbour, 12 m below sea level.	54° 32.80′ N 18° 36.20′ E
GN		Station located 19 km to the North-West of the Vistula estuary, 37 m below sea level.	54° 31.70′ N 18° 50.30′ E
ME		Station located about 1 km away from the coast, 4 m below sea level.	54° 36.44′ N 18° 31.59′ E

Five-centimetre-thick layers of sediment were collected using a core sampler. Samples were frozen until the time of analysis and subsequently lyophilized. The following physicochemical properties were determined in the sediment samples: wetness (W), loss-on-ignition (LOI), total carbon (TC), organic carbon (OC), black carbon (BC) and granulometric composition.

## Methods

### Physicochemical Properties

The wetness of sediments was determined by measuring the mass of wet sediment and then the dry mass after an approximately 24-h drying session at 105 °C. Granulometric analysis was performed by sieving dry sediment through a set of metal sieves of the following net dimensions: 2.00, 1.00, 0.5, 0.125, 0.063 mm. Loss-on-ignition was determined as a change of dry sediment mass after igniting at 550 °C until constant mass was reached (Heiri et al. 2001). All carbon types were determined using a PerkinElmer CHNS/O Analyzer through combustion of a dry sediment sample. Organic carbon was analysed using the chemothermal oxidation method (Gustafsson et al. 1997), the sample having previously been acidified in order to remove carbonates (Hedges and Stern 1984). When analysing black carbon, an additional stage was implemented following carbonate removal and consisted of ignition at 375 °C for 18 h with constant air flow.

### Alkylphenols

In order to assay alkylphenols in sediment samples, the following reagents were used: water, acetonitrile, methanol HPLC grade (Merck), chloric acid (VII) and ammonium acetate (analytically pure) (POCh), and high grade standards (97 %) of the analysed alkylphenols such as 4-nonylphenol and 4-*tert*-octylphenol (SIGMA-Aldrich). For the determination of physicochemical properties, a solution of 1 M hydrochloric acid and deionised water was used.

Lyophilised sediment samples (2 g) for alkylphenols assay were extracted twice for 15 min using 5 cm^3^ of a 30:70 mixture of deionised water and methanol. The obtained extractions were purified on SPE C18 columns using a method developed by Nunez et al. (2007). Elution was carried out three times with 1 cm^3^ of methanol and 1 cm^3^ of acetonitrile. The extracts were then dried by evaporation using a rotary evaporator and reconstituted in 200 μdm^3^ of acetonitrile.

The final analysis of alkylphenols in sediments was conducted using a high-performance liquid chromatograph Dionex UltiMate 3000 with a fluorescence detector (excitation *λ* = 275 nm, emission *λ* = 300 nm) and a Thermo Scientific HYPERSIL GOLD C18 PAH chromatography column (250 × 4.6 mm, 5 μm) in the mobile phase programme (water/acetonitrile) in gradient conditions.

### Validation Parameters

The linear correlation coefficient r in the analytical curves of working solutions with concentrations of 10, 25, 50, 75 and 100 ng cm^−3^ was higher than 0.999. The limit of quantification (LOQ) was 0.08 ng · g^−1^ dw for 4-nonylphenol and 4-*tert*-octylphenol. The background value was below quantification level. Mean recovery of both studied compounds in a sediment sample with a known amount of the standard was 94 and 81 % for 4-*tert*-octylphenol and 4-nonylphenol, respectively. Precision, expressed by the relative standard deviation coefficient (RSD) for five repetitions of the same sample, was below 8 % for each of the compounds.

### Statistical Analysis and Data Normalisation

The statistical analysis and a visual representation of the obtained results were carried out using the STATISTICA 10 programme by Stat Soft. The normality of data distribution was checked using the Shapiro-Wilk test (*p* < 0.05). Most results were of a non-parametric nature. The dependences were determined using the Spearman’s Rho correlation coefficient, adopting a confidence interval of 95 %.

NP and OP concentrations were normalised to the smallest fraction of sediment (Ø < 0.063 mm) using the following formula (Beldowski and Pempkowiak 2007):

$$ {\mathrm{APs}}_{\phi}=\frac{\left[\mathrm{APs}\right]}{10^{-2}\circ \phi <0.063\mathrm{mm}} $$

APs_*Ø*_: concentration of alkylphenols normalised to the smallest sediment fraction
APs: alkylphenol concentration
*Ø*: diameter of sediment particles

## Results and Discussion

### 4-*tert*-octylphenol and 4-nonylphenol Concentrations

**Fig. 2:**
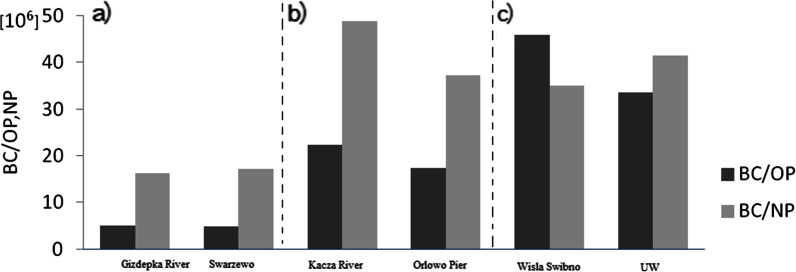
Proportions of black carbon (BC) to 4-nonylphenol (NP) and 4-*tert*-octylphenol (OP) in sediments from river stations and stations situated close to river outlets: **a** Gizdepka River (ST6): Swarzewo (ST7), **b** Kacza River (ST4): Orlowo Molo (ST3), **c** Vistula Swibno (ST1): UW

The mean value for 4-nonylphenol concentrations determined in the sediments of the Gulf of Gdansk amounted to 6.96 ng g^−1^ dw (Table 2), and this is comparable to concentrations obtained in other coastal areas of Europe. In 2003, Jonkers et al. found a mean NP concentration of 5.6 ng g^−1^ dw in the Scheldt and Rhine river estuaries (Holland), while similar values were found in sediment from Barcelona harbour (Spain) (9.25 ng g^−1^ dw) (Petrovic et al. 2002). However, the mean NP concentration in sediment from the German coast (North Sea) amounted to 32.5 ng g^−1^ dw (Bester et al. 2001). NP concentration levels in Asia were even higher than in Europe, reaching 64.5 ng g^−1^ dw in the sediments of the Sea of Japan (Hong and Shin 2009) and as much as 1.22 μg g^−1^ dw in sediments from the Yundang Lagoon in China (Zhang et al. 2011). In Jamaica Bay, near New York (USA), sediments exposed to pollution through petroleum recovery were characterised by average NP concentrations that were over 100-fold higher (846 ng g^−1^ dw) (Lee Ferguson et al. 2001) than those found in the sediments of the Gulf of Gdansk (Fig. 2).

**Table 2:** Characterisation of 4-nonylphenol and 4-*tert*-octylphenol concentration in sediment samples collected in river estuaries (A), at shallow coastal stations (B) and at deeper open water stations (C)

Sampling station/variable		4-nonylphenol [ng∙g^−1^ dw]	4-*tert*-octylphenol [ng g^−1^ dw]
A. Rivers			
		Min–max	Min–max
ST6	Gizdepka River	<LOQ—4.93	0.55–47.12
ST2	Kacza River	<LOQ—2.21	<LOQ—8.51
ST1	Vistula Swibno	<LOQ—4.44	<LOQ—23.43
*n*		18	18
*x* ± SD		2.26 ± 1.63	7.58 ± 13.43
Median		0.63	2.71
B. Coastal station			
ST7	Swarzewo	<LOQ—3.13	0.5–48.88
ST5	Mechelinki	<LOQ—2.32	0.14–38.72
ST4	Gdynia Seaside Boulevard	<LOQ—13.56	<LOQ—18.74
ST3	Orlowo	<LOQ—2.21	0.16–14.90
*n*		24	24
*x* ± SD		2.35 ± 3.38	9.57 ± 13.45
Median		1.34	3.24
C. Open water stations (depths over 4 m)			
ME		<LOQ—0.23	0.15–2.45
SP		<LOQ—2.66	1.02–11.75
GDY		<LOQ—4.24	0.34–20.47
GN		0.08–3.95	1.77–17.89
UW		<LOQ—249.08	2.61–18.44
*n*		30	30
*x* ± SD		12.58 ± 52.83	5.30 ± 5.51
Median		1.06	2.61

*n* sampling number, *x* mean value, *SD* standard deviation, *LOQ* limit of quantification, LOQ = 0.08 ng g^−1^ dw

Mean OP concentration in the sediments of the Gulf of Gdansk amounted to 7.46 ng g^−1^ dw (Table 2) and was lower than in other regions of the world. Estimated OP levels in sediments from the Thermaic Gulf in Greece were slightly higher at 10.3 ng g^−1^ dw (Arditsoglou and Voutsa 2012), but in sediments from the Mediterranean coast of Spain, OP concentrations were over eight times higher than 61 ng g^−1^ dw (Petrovic et al. 2002). As was the case with NP, the concentrations of OP were much higher in the sediments of South-East Asia than in the Gulf of Gdansk. Bottom sediments from Masan Bay in South Korea were characterised by mean OP concentrations of 91.5 ng g^−1^ dw (Khim et al. 1999), and equally high concentrations were discovered in bottom sediments on the coast of Taiwan (Cheng et al. 2006).

### Possible Sources of Alkylphenols in Different Seasons

On the basis of the obtained results, it was found that rivers are the main transportation route of hydrophobic pollutants related to black carbon into estuaries and coastal areas (Table 2). The same relationship is described by Mannino and Harvey (2004) with respect to the region of Chesapeake Bay by the Delaware estuary. The assays in the Gulf of Gdansk have shown that the relations of black carbon concentrations to 4-*tert*-octylphenol and 4-nonylphenol concentrations are similar in sediment samples both from river estuaries and stations located close to the outlets of small rivers (Fig. 2).

In each case, the revealed similarities indicated the same source of black carbon on which alkylphenols were sorbed. BC is a component of a suspension transported by rivers into seas and oceans (Mitra et al. 2002). Another dependency was indicated by the BC/NP ratios at the Vistula estuary station (ST1) and the UW station, located in the same area but further out to sea. The sediment from the latter station (UW) had BC/NP ratios higher than the other stations and the highest NP concentrations (249.08 ng g^−1^ dw). The differences resulted from the type of sediment (silt sediment); the depth of the station; and the highest content of total carbon (TC), organic carbon (OC) and black carbon (BC) (Table 3).

**Table 3:** Characterisation of physicochemical properties of surface sediments collected in river estuaries (A), in the coastal zone (B) and at stations located some distance away from the coast below the 4-m isobath (C)

Sampling station/variable		W [%]	LOI [%]	TC [%]	OC [%]	BC [%]	Type of sediment
Min–max							
A. Rivers							
ST6	Gizdepka River	12.6–26.1	0.3–0.8	0.087–0.455	0.017–0.580	0.007–0.035	Medium sand
ST2	Kacza River	12.5–17.8	0.5–21.6	0.170–0.515	0.040–0.226	0.068–0.087	Pebbles
ST1	Vistula Swibno	18.0–22.2	0.3–6.2	0.140–0.301	0.051–0.158	0.027–0.047	Silt
B. Coastal station							
ST7	Swarzewo	20.3–30.4	0.4–2.7	0.152–0.782	0.033–0.568	0.003–0.071	Medium sand
ST5	Mechelinki	12.8–21.6	0.1–0.5	0.156–0.603	0.019–0.053	0.025–0.091	Coarse sand
ST4	Gdynia Seaside Boulevard	7.0–18.5	0.3–0.8	0.133–0.610	0.017–0.070	0.002–0.097	Coarse sand
ST3	Orlowo Pier	16.3–19.9	0.2–6.1	0.070–0.249	0.006–0.065	0.001–0.062	Medium sand
C. Station below 4 m depth							
ME		13.9–21.7	0.4–13.5	0.102–0.336	0.047–0.107	0.008–0.047	Medium sand
SP		16.1–23.4	0.3–1.1	0.008–0.402	0.065–0.364	0.013–0.030	Medium sand
GDY		14.7–61.8	0.4–11.1	0.142–3.189	0.092–2.577	0.002–0.252	Medium sand
GN		17.0–34.0	0.4–2.7	0.235–2.027	0.121–1.450	0.009–0.215	Silt
UW		17.1–63.0	0.4–16.6	0.101–3.331	0.070–2.742	0.010–0.271	Silt

*W* wetness, *LOI* loss of ignitron, *TC* total carbon, *BC* black car bon, *OC* organic carbon

**Fig. 3:**
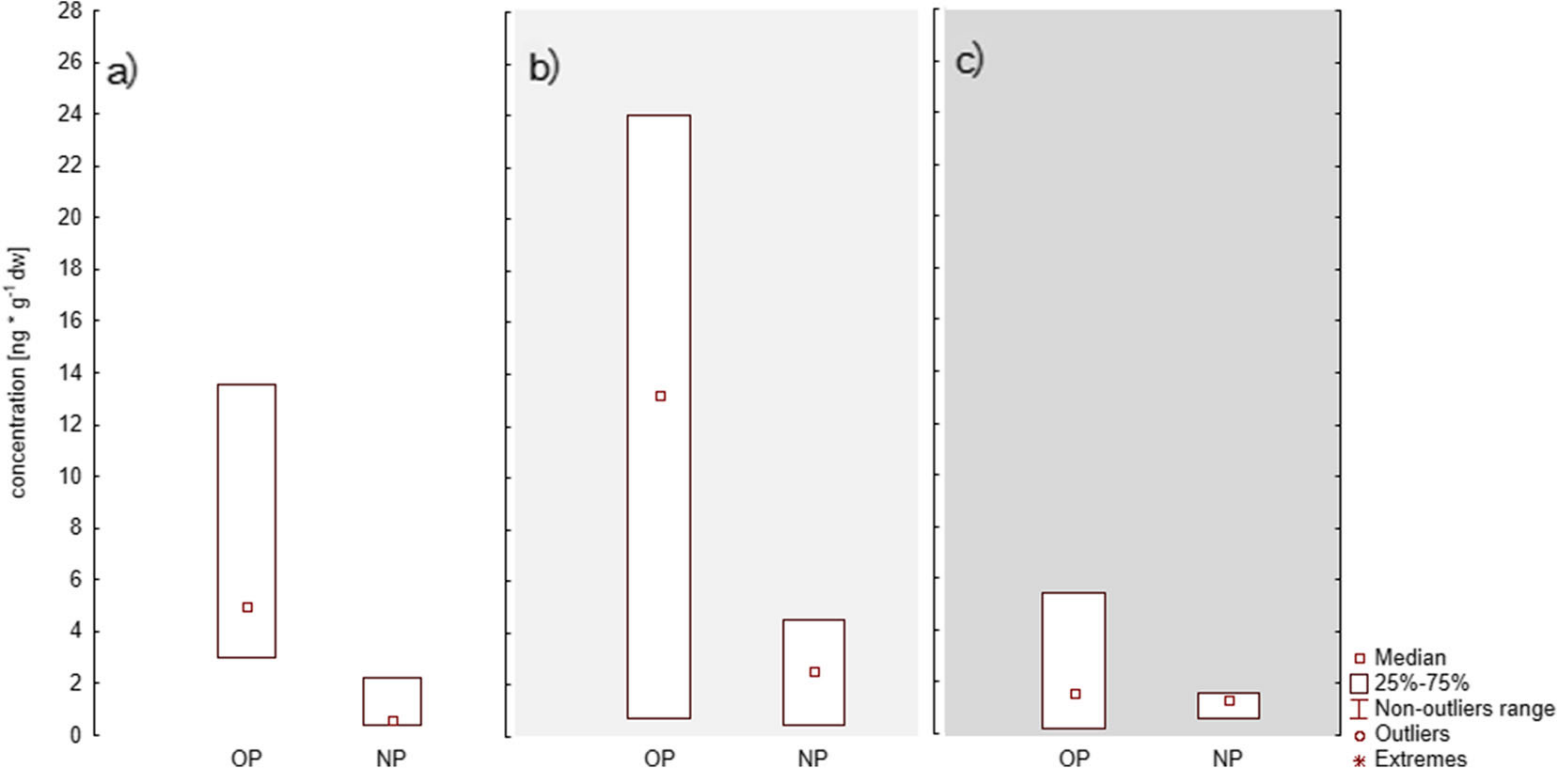
Seasonal changes in 4-nonylphenol (NP) and 4-*tert*-octylphenol (OP) in sediments from river stations ST1, ST2, ST6 in **a** spring, **b** summer and **c** autumn

In the case of 4-*tert*-octylphenol, its mean concentrations in sediment from estuary stations (A) (7.58 ng g^−1^dw) were higher than mean concentrations found in sediments from open water stations (C) (5.31 ng g^−1^ dw) (Table 3). Similar results were obtained by Zhang et al. (2011), indicating that a significant load of surface active agents containing NP and OP was introduced into the sea via river transportation. In summer, the differences in OP and NP concentrations between the stations of the coastal zone (B) and the open water stations (C) were even more pronounced due to higher water temperature. In that period, the breakdown of alkylphenol etoxylates in the coastal zone occurs more rapidly and can result in an increase in NP and OP concentrations in sediment collected in river estuaries. On the other hand, in deeper waters, where the temperature is lower, etoxylate decomposition may be slower or limited. This is confirmed by results found in literature from around the world. Manzano et al. (1999) discovered that alkylphenol etoxylate breakdown takes place more rapidly at 22.5 °C than at 13 °C. This was also observed during tests carried out in the Gulf of Gdansk (Fig. 3) as the highest OP and NP concentrations occurred in summer (Fig. 3b). In spring (Fig. 3a), there was probably a second factor which effected an increase in alkylphenol concentrations in river estuary sediment, namely the increased river dynamics of that season causing the resuspension of organic matter containing NP and OP.

**Fig. 4:**
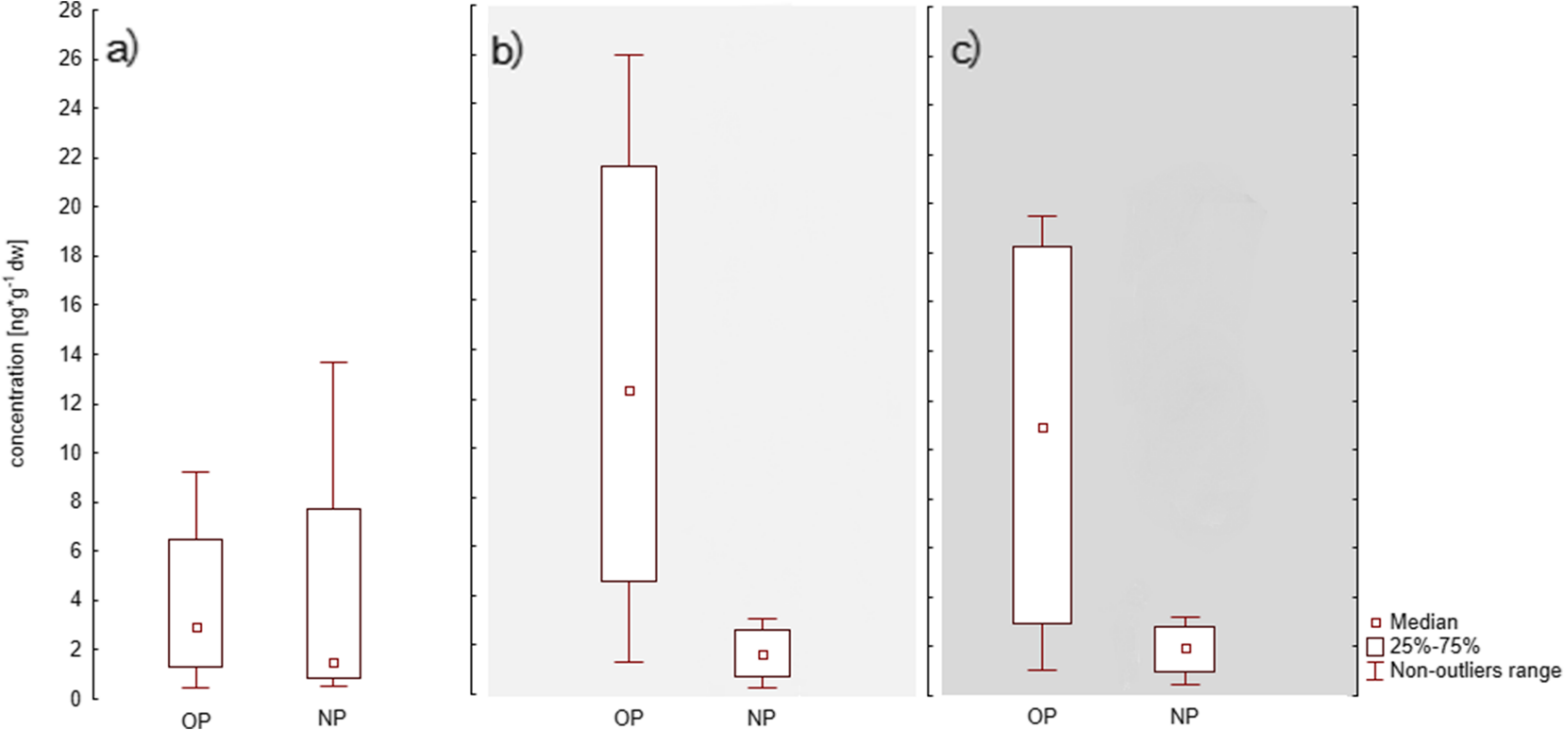
Seasonal changes in 4-nonylphenol (NP) and 4-*tert*-octylphenol (OP) in sediments from coastal stations ST3, ST4, ST5, ST7 in **a** spring, **b** summer and **c** autumn

At coastal stations (Fig. 4), increased alkylphenol concentrations were influenced by the proximity to beaches and public spaces as well as by high temperature. At those sites, high concentrations of 4-*tert*-octylphenol were observed (Fig. 4b), and this was probably related to the widespread use of OP in suncreams or other cosmetics and synthetic materials used by tourists.

**Fig. 5:**
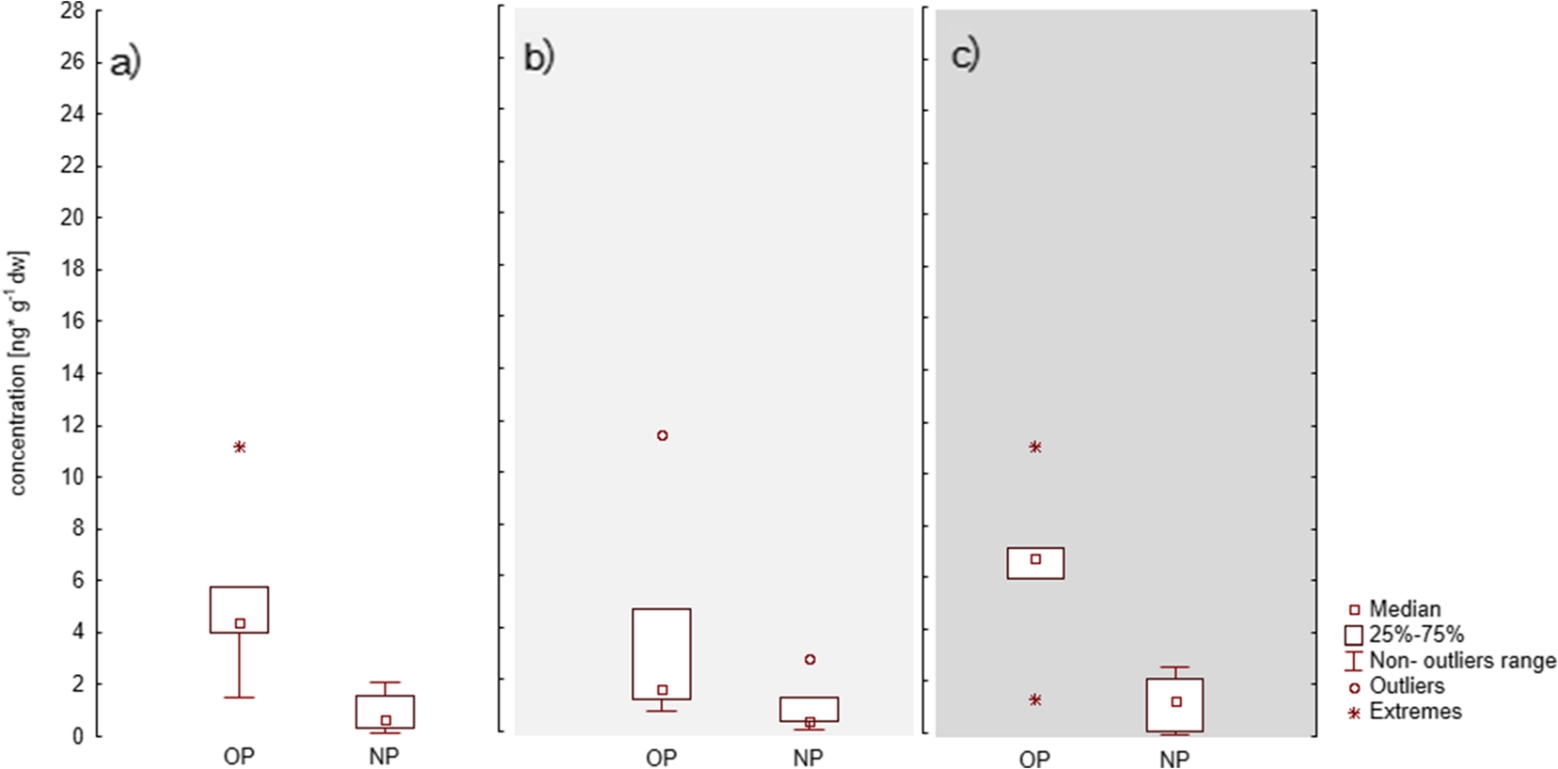
Seasonal changes in 4-nonylphenol (NP) and 4-*tert*-octylphenol (OP) in sediments from open water stations located below the 4-m isobath ME, SP, GDY, GN, UW in **a** spring, **b** summer and **c** autumn

At open water stations below the 4-m isobath (Fig. 5), the highest concentrations of OP and NP were observed in autumn, and this has to be linked with alkylphenols introduced to surface sediments with suspension about 6 months previously, in springtime (Fig. 5c).

### Black Carbon Influence

The dependency between NP and OP concentrations within the smallest sediment fraction in samples from open water stations, located at depths of over 4 m, and the BC/OC ratio was indicated by high correlation coefficients (NP *r* = 0.70, *p* = 0.02; OP *r* = 0.75, *p* = 0.02). In terms of the occurrence of seasonal changes in alkylphenol concentrations, a strong correlation was observed only in sediments collected in autumn (*r* = 0.89 for NP and *r* = 0.94 for OP, *p* < 0.03).

Owing to the fact that autumn is a time when fossil fuel combustion increases in thermal-electric power stations and individual households, it should be expected that atmospheric deposition plays at that time a significant role in delivering BC, accompanied by alkylphenols, to surface waters. Another way in which BC enters the environment is through biomass combustion. Although most BC is deposited close to emission sites, its transportation via rivers and the atmosphere is also possible (Mannino and Harvey 2004). This is confirmed by results presented in a publication prepared by Lewandowska et al. (2012), which show that alkylphenols can be found in small aerosols (PM1 and PM2), closely correlated with the presence of BC. The highest OP and NP concentrations in small aerosols were determined in autumn and winter, that is, during the heating season. In these seasons, additionally, it is dominated by air mass paths from sectors: S and SW caring heating contaminations and BC from surrounding villages (Staniszewska et al. 2013).

The results obtained in the Gulf of Gdansk are also confirmed by publications where the authors indicate the possibility of atmospheric transportation and deposition of black carbon and alkylphenol into surface waters (Xie et al. 2006). Black carbon is the most important aerosol component, influencing the sorption of hydrophobic pollutants deposited on the surface of sediments (Staniszewska et al. 2011). Owing to the dependency between NP and OP and the BC/OC ratio within the smallest sediment particles, atmospheric deposition can be considered a very likely source of alkylphenols.

### Sorptive Properties of 4-nonylphenol and 4-*tert*-octylphenol

4-*tert*-octylphenol had higher concentrations in sediments than 4-nonylphenol. This was undoubtedly influenced by the widespread use of OP in industry and the lack of legal restrictions in the EU. OPs are used mainly as additives to synthetic materials, serving as glue or binding agents during emulsion polymerisation. Octylphenol etoxylates are used in the production of textiles covered with a thin polymer film that makes the material more resistant to water, dust and light, and gives it a glossy look (e.g. leather). They are also used in the production of cosmetics (HELCOM 2011). An increased amount of surface active agents can alter the sorptive properties of sediments, which in turn can cause changes in the distribution of various hydrophobic substances (Yang et al. 2011).

**Fig. 6:**
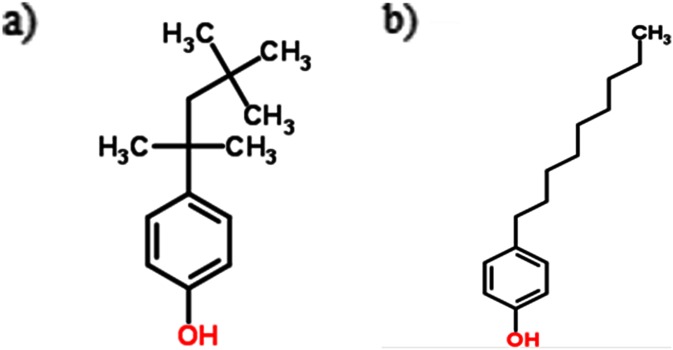
Structural composition of **a** OP and **b** NP

Another factor influencing the occurrence of higher concentrations of OP than NP is OP’s greater residence time in the environment. OP has a different, more branched out, hydrocarbon chain structure than NP (Pignatello 1998) (Fig. 6). Owing to that, its residence time in the environment is longer as the decomposition process is slower. The sediments of the Gulf of Gdansk are often short in oxygen, which can result in the breakdown of alkylphenol etoxylates becoming decelerated (Falkowska et al. 1993). At stations situated in deeper water, OP concentrations were nevertheless lower than in the coastal area. Despite its long residence time, OP was not strongly connected to sediment. This is confirmed by a lack of correlation between OP concentration and carbon forms (TC, OC and BC). However, such correlations were observed for 4-nonylphenol (0.76 > *r* > 0.96, *p* < 0.03). According to Heinis et al. (1999), the half-life time for 4-nonylphenol in anaerobic conditions is 66 days, while 401 days are required for a complete breakdown of this compound and 95 % of its removal. When oxygen is involved, the decomposition of 4-nonylphenol occurs more rapidly, with nearly 50 % of this compound decomposing within 10 days (Ying et al. 2003). Having a linear hydrocarbon chain structure, 4-nonylphenol becomes more easily sorbed onto sediment particles (Fig. 6). This compound is characterised by higher values of *K*_o/c_ coefficient (38.90 thousand dm^3^ · kg^−1^) and *K*_o/w_ coefficient (4.48) than is the case with 4-*tert*-octylphenol (*K*_o/w_ = 4.12, *K*_o/c_ = 18.20 thousand dm^3^ · kg^−1^).

## Risk to the Marine Environment

Alkylphenols accumulated in sediments may have a negative effect on benthic organisms, especially silt-eating ones, at a low level of the trophic chain. According to US EPA, the indicator of alkylphenol noxiousness for benthic organisms is PNEC (predicted no effect concentration), which signifies the concentration level below which there are no harmful consequences for the environment. 4-*tert*-octylphenol concentrations exceeded the EPA-adopted PNEC value by 8 % and 4-nonylphenol by 3 % at 34 ng g^−1^ dw for OP and 170 ng g^−1^ dw for NP. It can therefore be said that the sediments of the Gulf of Gdansk are not badly polluted with alkylphenols. A greater number of exceedances were observed in sediments from the Gulf of Finland, the Danish straits and the Northern part of Baltic Proper (HELCOM 2010).

## Summary

The increasingly widespread use of alkylphenols in European industry has led to these compounds being found in measurable concentration levels in surface sediments of the Gulf of Gdansk. 4-nonylphenol and 4-*tert*-octylphenol concentrations were comparable to other coastal regions of Europe, but much lower, even by several orders of magnitude, than in sea sediment from the regions of South-East Asia. Increased concentrations in sediments, mainly in the coastal zone, were found particularly in summertime, when at higher water temperatures the decomposition of alkylphenol precursors (etoxylates) occurred more rapidly. Well-developed tourism and increased recreation in the coastal area, especially during summer, result in a higher consumption of products containing alkylphenols. In spring, high 4-nonylphenol and 4-*tert*-octylphenol concentrations arrived in the coastal zone with meltwater.

The obtained results have shown that rivers and surface run-off are the main sources of alkylphenols in coastal sediments of the Gulf of Gdansk, whereas the role of the atmosphere as a means of alkylphenol transportation over the sea increases in significance mainly in autumn. The high correlation coefficients found between NP and OP concentrations and the BC/OC ratio confirm the significance of black carbon originating from combustion processes on land as a factor of alkylphenol distribution in the environment.
